# Dihydropashanone Isolated from *Lindera erythrocarpa*, a Potential Natural Product for the Treatment of Neurodegenerative Diseases

**DOI:** 10.3390/ijms25052545

**Published:** 2024-02-22

**Authors:** Zhiming Liu, Chi-Su Yoon, Hwan Lee, Hyeong-Kyu Lee, Dong-Sung Lee

**Affiliations:** 1 College of Pharmacy, Chosun University, Dong-gu, Gwangju 61452, Republic of Korea; lzmqust@126.com (Z.L.); ghksdldi123@hanmail.net (H.L.); 2 Institute of Pharmaceutical Research and Development, College of Pharmacy, Wonkwang University, Iksan 54538, Republic of Korea; ycs1991@naver.com; 3 Natural Medicine Research Center, Korea Research Institute of Bioscience and Biotechnology (KRIBB), Cheongju 28116, Republic of Korea; brightjem6178@gmail.com

**Keywords:** dihydropashanone, neuroprotective effects, Nrf2, NF-κB

## Abstract

*Lindera erythrocarpa*, a flowering plant native to eastern Asia, has been reported to have neuroprotective activity. However, reports on the specific bioactive compounds in *L. erythrocarpa* are finite. The aim of this study was to investigate the anti-neuroinflammatory and neuroprotective effects of the compounds isolated from *L. erythrocarpa*. Dihydropashanone, a compound isolated from *L. erythrocarpa* extract, was found to have protected mouse hippocampus HT22 cells from glutamate-induced cell death. The antioxidant and anti-inflammatory properties of dihydropashanone in mouse microglial BV2 and HT22 cells were explored in this study. The results reveal that dihydropashanone inhibits lipopolysaccharide-induced inflammatory response and suppresses the activation of nuclear factor (NF)-κB in BV2 cells. In addition, dihydropashanone reduced the buildup of reactive oxygen species in HT22 cells and induced activation of the nuclear factor E2-related factor 2 (Nrf2)/heme oxygenase (HO)-1 signaling pathway in BV2 and HT22 cells. Our results suggest that dihydropashanone reduces neuroinflammation by decreasing NF-κB activation in microglia cells and protects neurons from oxidative stress via the activation of the Nrf2/HO-1 pathway. Thus, our data suggest that dihydropashanone offers a broad range of applications in the treatment of neurodegenerative illnesses.

## 1. Introduction

Neurodegenerative disorders, such as Alzheimer’s disease (AD), Parkinson’s disease (PD), and multiple sclerosis, are defined by neuronal destruction [1]. The progression of neurodegenerative diseases is generally accompanied by mitochondrial dysfunction, which leads to the excessive generation of reactive oxygen species (ROS) and to oxidative stress in the central nervous system (CNS) [2]. ROS-mediated oxidative damage can lead to serious damage to cellular structures [3] and can enhance the release of proinflammatory mediator proteins, such as inducible nitric oxide synthase (iNOS) and cyclooxygenase-2 (COX-2), these proteins can regulate the secretion of nitric oxide (NO) and prostaglandin E2 (PGE_2_) [4], thus leading to neuroinflammation [5]. Microglia are crucial immune cells in neuroinflammation and change their phenotypic and function in response to environmental signals [6]. When stimulated by endotoxins, such as lipopolysaccharide (LPS), microglia acquire the characteristic M1 phenotype and exhibit proinflammatory effects [7]. Therefore, LPS-induced M1 phenotype microglia promote to the exacerbation of oxidative stress and inflammation in the nervous system [8]. The M1 microglia cause the release of various inflammatory markers, including tumor necrosis factor-α (TNF-α) and interleukin-6 (IL-6), which may directly damage the CNS and lead to neurodegenerative diseases [9]. Recently, increasing attention has been paid to the identification of novel natural agents that can control microglial activation to treat or prevent neuroinflammation [10,11,12]. Functional neuronal cell loss is a significant cause of neurodegenerative illnesses, and glutamate is an excitatory neurotransmitter that plays a key role in synaptic plasticity in the brain [13]. However, glutamate imbalance (excessively high levels) can damage cellular components, including mitochondria, and promote ROS production, leading to neurotoxicity and neuronal cell damage [14]. Thus, glutamate plays a significant role in the pathogenic process of different neurodegenerative disorders, especially AD and PD [15,16]. Therefore, inhibiting oxidative stress caused by glutamate is an important strategy for protecting neurons.

*Lindera erythrocarpa* (Lauraceae) is widespread in South Korea, Japan, and China. In our earlier study, 18 compounds were identified in *L. erythrocarpa*, including dihydropashanone [17]. Dihydropashanone was found to inhibit the excessive production of LPS-induced NO in both BV2 and RAW264.7 cells [18]. However, dihydropashanone has only been confirmed to inhibit NO production in BV2 cells [18] and no additional mechanistic studies related to inflammation regulation have been conducted. Furthermore, the primary aim of this study is to show, for the first time, the neuroprotective effects of 18 compounds in hippocampal HT22 cells. Therefore, this study shows the protective effects of the 18 compounds isolated from *L. erythrocarpa* against glutamate-induced HT22 cell damage and verifies the neuroprotective mechanism of dihydropashanone. In addition, the effects of dihydropashanone in the inhibition of LPS-mediated inflammatory response and its mechanisms in BV2 cells was examined.

## 2. Results

### 2.1. Protective Effects of the 18 Compounds Isolated from L. erythrocarpa on HT22 Cells

In our previous report, the isolation of 18 compounds from *L*. *erythrocarpa* extract was described [17]. The 18 compounds are as follows: kanakugiol (**1**), 2′-hydroxy-3′,4′,6′-trimethoxychalcone (**2**), pashanone (**3**), kanakugin (**4**), 5-hydroxy-7,8-dimethoxyflavanone (**5**), onysilin (**6**), dihydropashanone (**7**), 2-hydroxy-3′,4′,6′-trimethoxydihydrochalcone (**8**), avicularin (**9**), afzelin (**10**), avicularin-acetate (**11**), quercitrin (**12**), linderone (**13**), methyllinderone (**14**), methyllucidone (**15**), linderaspirone A (**16**), bi-linderone (**17**) and demethoxy-bi-linderone (**18**).

**Figure 1:**
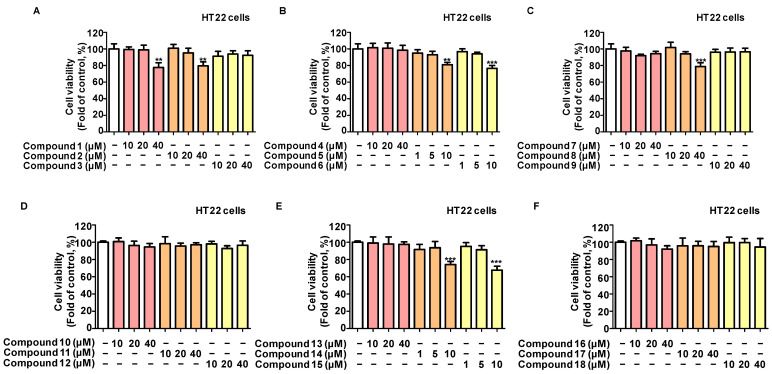
Cytotoxicity of the 18 compounds on HT22 cells. HT22 cells were treated with varying doses of the compounds for 24 h, and cell viability (**A**–**F**) was assessed using the MTT test. Values are reported as % of control and presented as the mean ± standard deviation (SD) of at least three independent experiments. ** *p* < 0.01, *** *p* < 0.001 vs. control group.

To determine if these 18 chemicals extracted from *L. erythrocarpa* protect against neural cell damage, HT22 mouse hippocampus cells were treated with glutamate in the presence or absence of different doses of these compounds. An MTT test was used to assess the cytotoxicity of all 18 components (Figure 1). Non-toxic concentrations of each component were determined and applied in the following experiments.

**Figure 2:**
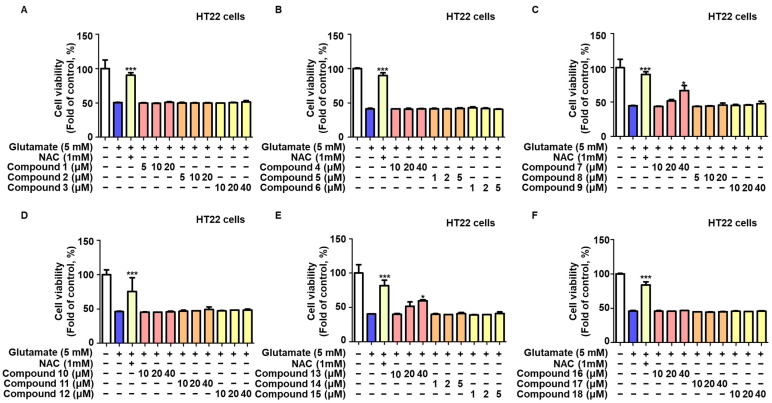
Protective effects of 18 compounds on glutamate-induced HT22 cell toxicity. HT22 Cells were pretreated with various doses of the compounds for 2 h, then treated with glutamate for 24 h, and cell viability (**A**–**F**) was determined using the MTT test. Values are reported as % of control and presented as the mean ± SD of at least three independent experiments. * *p* < 0.05, *** *p* < 0.001 vs. glutamate group.

Figure 2 shows that glutamate reduced HT22 cell viability by around 50% compared with the control group. Solutions of 40 µM of compounds **7** (dihydropashanone) and **13** (linderone) showed significant protective effects on glutamate-induced neuronal cell damage. NAC was used as a positive control.

### 2.2. Effects of Dihydropashanone on Inflammatory Response in BV2 Cells

**Figure 3:**
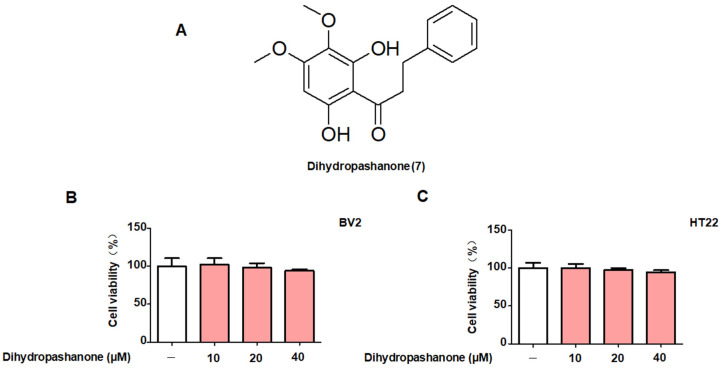
Cytotoxicity of dihydropashanone in BV2 and HT22 cells. (**A**) Structure of dihydropashanone. (**B**) BV2 and (**C**) HT22 cells were treated with dihydropashanone (10–40 μM) and cell viability was assessed using the MTT test. Values are reported as % of control and presented as the mean ± SD of at least three independent experiments.

Based on the results of the toxicity, compound **7**, dihydropashanone (Figure 3A), was selected for further mechanism study, the reporting of which is a first for this compound. First, we conducted MTT assays in order to reconfirm the non-toxic concentrations of dihydropashanone in BV2 and HT22 cells. BV2 and HT22 cells were treated with compound **7** (10–40 μM) for 24 h. The results reveal that the viability of cells treated with various doses of dihydropashanone was equivalent to that of the control group cells (Figure 3B,C), indicating that compound **7** exhibited no cytotoxic effect on BV2 and HT22 cells in the concentration range tested.

**Figure 4:**
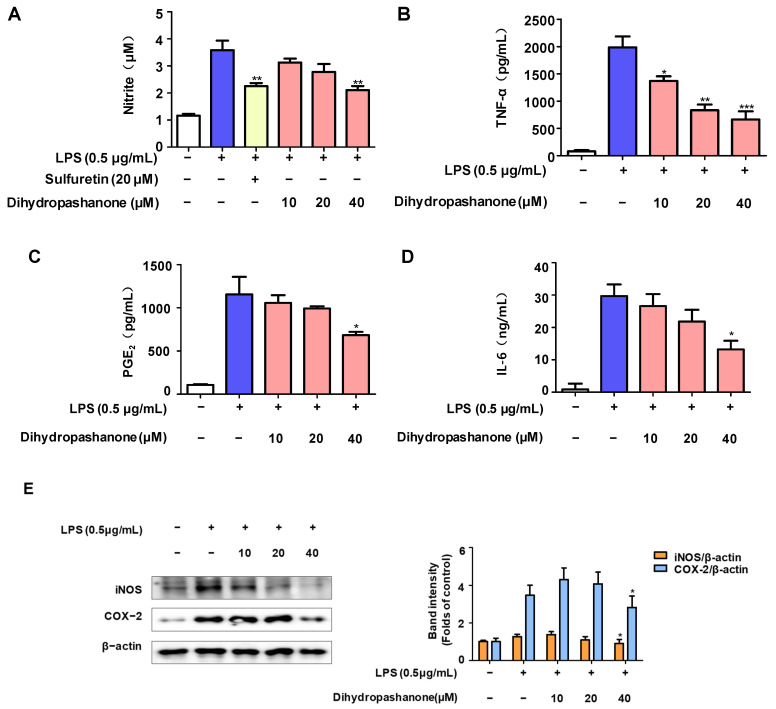
Anti-inflammatory effects of dihydropashanone in LPS-induced BV2 cells. BV2 cells were pretreated with dihydropashanone for 2 h, and then exposed to LPS for 24 h. (**A**) Nitrite was determined using Griess assay. Sulfuretin was used as a positive control. TNF-α (**B**), PGE_2_ (**C**), and IL-6 (**D**) were determined using enzyme-linked immunosorbent assay (ELISA), and iNOS and COX-2 expression (**E**) were determined using Western blot analysis. Values are presented as the mean ± SD of at least three independent experiments. * *p* < 0.05, ** *p* < 0.01, *** *p* < 0.001 vs. LPS group.

Next, dihydropashanone’s anti-inflammatory properties were investigated on LPS-induced BV2 microglial cells. BV2 cells were exposed to different concentrations of dihydropashanone (10–40 μM) for 2 h. Then, LPS was used to induce inflammation. Figure 4 demonstrates that LPS stimulation increased the production of inflammatory factors in BV2 cells, including nitrite, TNF-α, IL-6, and PGE_2_ in BV2 cells. Pretreatment of cells with dihydropashanone significantly inhibited the production of inflammatory factors. Our results show that 40 µM of dihydropashanone induced the maximum anti-inflammatory effect (Figure 4A–D). In addition, 40 µM of dihydropashanone significantly blocked the increased expression of inflammatory protein mediators, iNOS and COX-2, in LPS-induced BV2 cells (Figure 4E).

### 2.3. Effects of Dihydropashanone on the Regulation of NF-κB Pathway in BV2 Cells

NF-κB (p65) expression is a characteristic marker of inflammation. Therefore, we evaluated whether compound **7** inhibits LPS-induced stimulation of the NF-κB pathway. BV2 cells were co-treated with LPS for 30 min in the presence or absence of compound **7**.

**Figure 5:**
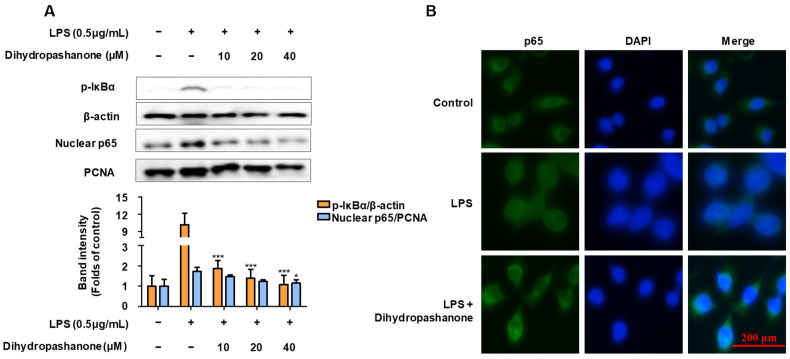
Inhibitory effect of dihydropashanone on NF-κB activation in LPS-induced BV2 cells. BV2 cells were pretreated with dihydropashanone for 2 h, and then exposed to LPS for 0.5 h. p-IκBα in cytoplasm and p65 in nucleus were determined using Western blot analysis (**A**). Nuclear translocation of p65 was analyzed using immunofluorescence assay (**B**). Values are presented as the mean ± SD of at least three independent experiments. * *p* < 0.05, *** *p* < 0.001 vs. LPS group.

A reagent kit was used to extract cytoplasmic and nuclear proteins. The phosphorylation of IκB-α, and the nuclear translocation of p65 were analyzed using Western blotting (Figure 5A). LPS treatment led to an increase in p-IκBα in the cytoplasm, and an increase in p65 expression in the nucleus. Dihydropashanone treatment significantly inhibited the activation of NF-κB nuclear translocation in LPS-induced BV2 cells. To observe the nuclear translocation of p65 more clearly, immunofluorescence assay was used to detect p65 in BV2 cells. As shown in Figure 5B, p65 was more abundant in the nuclei of LPS-induced cells compared with that in the control cells, whereas co-treatment with dihydropashanone inhibited the nuclear translocation of p65.

### 2.4. Effects of Dihydropashanone on Glutamate-Induced HT22 Cell Damage

**Figure 6:**
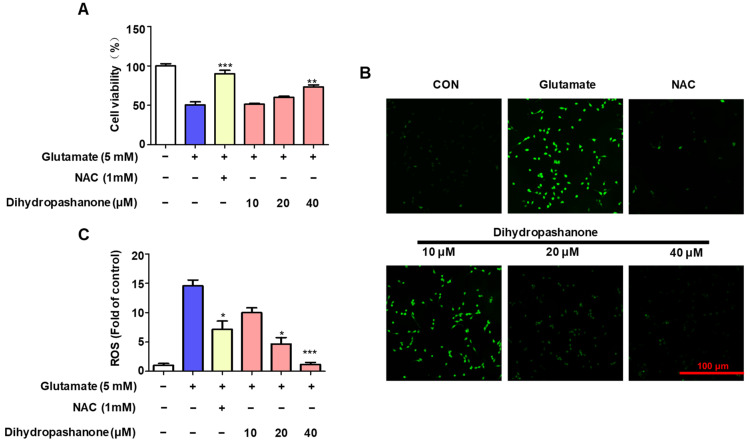
Antioxidant effect of dihydropashanone in glutamate-induced HT22 cells. HT22 cells were pretreated with different concentrations of dihydropashanone for 2 h, and then exposed to glutamate for 24 h. Cells viability was then tested using the MTT assay (**A**). Cells were pretreated with dihydropashanone for 2 h, and then exposed to glutamate for 8 h. ROS (**B**,**C**) levels were determined using the dichlorodihydrofluorescein diacetate (DCFDA) assay. Values are presented as the mean ± SD of at least three independent experiments. * *p* < 0.05, ** *p* < 0.01, *** *p* < 0.001 vs. glutamate group.

To examine the protective effect of dihydropashanone on glutamate-induced HT22 cell damage, HT22 cells were first treated with dihydropashanone (10–40 μM) for 2 h. Then, cells were treated with glutamate to promote cell death. The results show that, in the presence of glutamate, cell viability was reduced by approximately 50%; however, the co-treatment of cells with different concentrations of dihydropashanone weakened the glutamate-induced toxicity (Figure 6A). To investigate the antioxidant effects of dihydropashanone in glutamate-treated neural cells, its effect on intracellular ROS production was analyzed. HT22 cells produced ROS when treated with glutamate alone. However, dihydropashanone weakened the accumulation of intracellular ROS in a concentration-dependent manner. The results show that 40 µM of dihydropashanone achieved the maximum antioxidant effect (Figure 6B,C).

### 2.5. Effects of Dihydropashanone on Regulation of Nrf2/HO-1 Pathway

**Figure 7:**
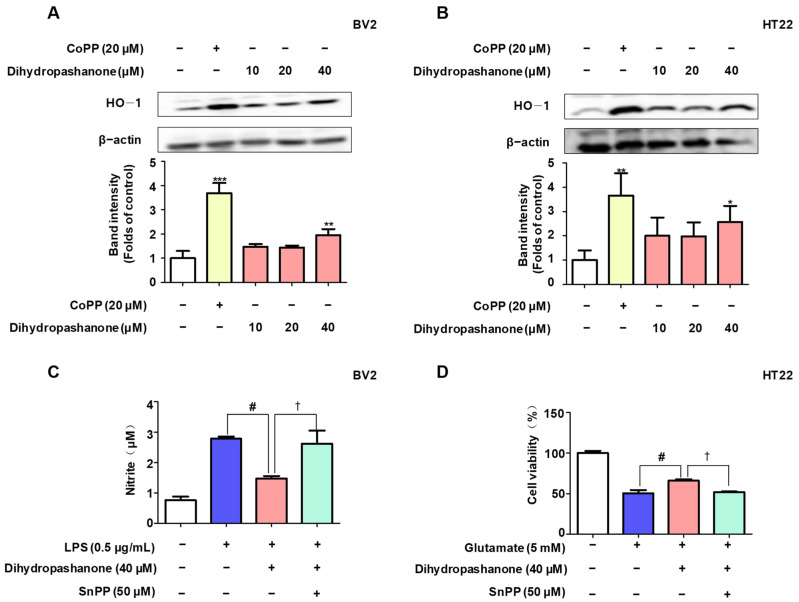
Dihydropashanone induced expression of HO-1 in BV2 and HT22 cells. Following treatment with dihydropashanone for 12 h, HO-1 expression was determined in BV2 (**A**) and HT22 cells (**B**) using Western blot analysis. CoPP was used as positive control. Cells were pretreated with dihydropashanone or SnPP for 2 h, and then exposed to LPS for 24 h. Nitrite (**C**) was determined using Griess assay. Cells were pretreated with dihydropashanone or SnPP for 2 h, and then exposed to glutamate for 24 h. Cell viability (**D**) was assessed using MTT assay. Values are presented as the mean ± SD of at least three independent experiments. * *p* < 0.05, ** *p* < 0.01, *** *p* < 0.001 vs. control group; # *p* < 0.05 vs. LPS or glutamate group; † *p* < 0.05 compared with LPS or glutamate with dihydropashanone-treated group.

To explore the mechanism behind the neuroprotective action of dihydropashanone against glutamate toxicity, the effect of dihydropashanone on the expression of the antioxidant enzyme HO-1 in cells was investigated. Dihydropashanone was used to treat BV2 and HT22 cells for 12 h. CoPP, an HO-1 agonist, was used as a positive control. Dihydropashanone treatment enhanced intracellular HO-1 expression in a concentration-dependent manner. (Figure 7A,B). To verify whether dihydropashanone exerts protective effects in the cells by upregulating HO-1 expression, SnPP, an HO-1 inhibitor, was used. The findings reveal that SnPP abrogated the protective effects of dihydropashanone on BV2 and HT22 cells. (Figure 7C,D).

**Figure 8:**
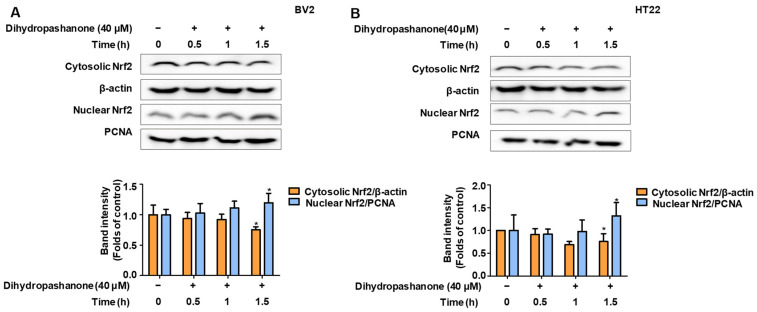
Dihydropashanone induced activation of Nrf2 in BV2 and HT22 cells. Following treatment with dihydropashanone, Nrf2 expression was determined in BV2 (**A**) and HT22 cells (**B**) at different time points. Values are presented as the mean ± SD of at least three independent experiments. * *p* < 0.05 vs. glutamate group.

Given that dihydropashanone induced HO-1 expression, we focused on Nrf2, an upstream HO-1 regulatory protein. Activation of Nrf2 signaling promotes the production of HO-1 through nuclear translocation. BV2 and HT22 cells were treated with 40 μM of dihydropashanone and Nrf2 levels in the nucleus and cytoplasm were detected at different time points. The results indicate that the nuclear Nrf2 levels increased significantly following 1.5 h of dihydropashanone treatment, whereas the level of Nrf2 in the cytoplasm decreased significantly (Figure 8A,B). The results show that 40 μM of dihydropashanone increases nuclear translocation of Nrf2 in BV2 and HT22 cells.

## 3. Discussion

Neurodegenerative diseases (such as AD) are important diseases that affect the elderly population. Natural products are regarded as effective means of combating neurodegenerative diseases [19,20,21,22]. *L. erythrocarpa* is a common plant species in eastern Asia. An earlier investigation revealed the isolation of various chemicals from *L. erythrocarpa* that may have anti-neuroinflammatory properties [17]. However, there has been little research into these substances’ neuroprotective properties. As a result, this study conducted a re-evaluation of the neuroprotective properties of chemicals extracted from *L. erythrocarpa.* The present study may provide additional options for treating neurodegenerative diseases.

First, the protective effects of the 18 compounds isolated from *L. erythrocarpa* were evaluated on neural cells. To produce toxicity in HT22 cells, large concentrations of glutamate were utilized, and the cells were co-cultured with the 18 compounds. The results show that only compounds **7** (dihydropashanone) and **13** (linderone) exhibited neuroprotective effects in the neural cells. Previous studies have demonstrated the anti-neuroinflammatory effects of linderone [18]. Therefore, we chose to focus on dihydropashanone for this study.

Microglia are important immune cells in the CNS. When exposed to LPS, microglia release inflammatory cytokines, leading to neuronal dysfunction in the brain and the development of neurodegenerative diseases [23]. Therefore, natural products that can inhibit the excessive production of inflammatory mediators by microglia may serve as potential therapeutic agents [24]. This study discovered that therapy with dihydropashanone greatly decreased the production of inflammatory components, including nitric oxide, in the damaged BV2 cells, and inhibited the expression of iNOS [25]. In addition, PGE_2_ is synthesized from arachidonic acid via COX-2 enzyme, which contributes to the progression of inflammation and pain [26]. Dihydropashanone also inhibits the overexpression of COX-2 and PGE_2_ in cells. Dihydropashanone significantly inhibits the production of inflammatory factor TNF-α and IL-6, which are affected by NF-κB activation [27]. The effect of dihydropashanone on the NF-κB signaling pathway was studied. LPS triggers the synthesis of neurotoxic cytokines and inflammatory mediators through an inflammatory cascade that includes NF-κB transcription factors [28]. Under normal conditions, p65 stably binds to IκB-α in the cytoplasm [29]. Following LPS induced IκB-α phosphorylation, p65 translocates to the nucleus and binds to targets on DNA, promoting the production of inflammatory factors [30]. In our study, LPS significantly induced phosphorylation of IκB-α. The level of nuclear p65 was elevated in the LPS-stimulated cells. Immunofluorescence assay demonstrated that p65 had a much higher nuclear abundance increase in LPS-treated cells. Dihydropashanone treatment significantly reduced the level of p-IκB-α in LPS-damaged cytoplasm. The nuclear translocation of p65 was also inhibited by dihydropashanone.

Glutamate is a key neurotransmitter in the brain. However, excessive levels of glutamate promotes ROS production in cells, damages mitochondria and DNA in neuronal cells, and causes neuronal cell damage [31]. Natural products with antioxidant properties neutralize ROS [32,33,34]. Our results show that glutamate significantly reduced HT22 cell viability by approximately 50%, whereas dihydropashanone reduced glutamate toxicity in a dose-dependent manner. Immunofluorescence assay results show that glutamate significantly increased intracellular ROS accumulation in HT22 cells, whereas ROS levels decreased significantly following treatment of the cells with dihydropashanone. In addition, ROS neutralization requires the expression of antioxidant enzymes. HO-1 is a phase II metabolic enzyme that has a decreasing impact, lowering ROS levels [35]. The findings reveal that dihydropashanone dramatically increases the expression of HO-1 in BV2 and HT22 cells. To verify whether dihydropashanone exerts antioxidant effects by regulating HO-1 expression, SnPP (HO-1 inhibitor) was used [36]. The findings indicate that SnPP inhibited the positive effects of dihydropashanone in BV2 and HT22 cells. These findings suggest that dihydropashanone protects cells by upregulating the expression of the antioxidant enzyme HO-1. Nrf2 is an upstream regulatory protein of HO-1. Activated Nrf2 translocates from the cytoplasm to the nucleus to promote the expression of various antioxidant enzymes, including HO-1 [37]. Given that dihydropashanone enhanced HO-1 expression, the effect of dihydropashanone on Nrf2 was tested in BV2 cells and HT22 cells at different time points. The results show that 40 of μM dihydropashanone significantly promoted the activation of Nrf2 after 1.5 h of treatment. In conclusion, dihydropashanone’s neuroprotective action against glutamate-induced cytotoxicity is mediated via the Nrf2/HO-1 pathway.

## 4. Materials and Methods

### 4.1. Materials

Dihydropashanone was isolated from the leaves of *L. erythrocarpa*. The leaves of *L. erythrocarpa* (1.5 kg) were ground and extracted by methanol, and partitioned with hexane, ethyl acetate, and *n*-butanol. The hexane fraction was further fractionated by silica gel and C18 column chromatography and purified by HPLC to obtain dihydropashanone (33 mg, yield 0.000022%). Detailed purification steps are described in our previous literature [17]. LPS and glutamate were obtained from Sigma-Aldrich (St. Louis, MO, USA). Cell culture reagents were purchased from Gibco BRL Co. (Grand Island, NY, USA).

### 4.2. Cell Culture and Cell Viability Assays

Mouse microglial BV2 and mouse hippocampal neuronal HT22 cells were purchased from ATCC. The culture condition of BV2 cells were RPMI medium supplemented with 10% (*v/v*) FBS. HT22 cells were cultured in DMEM supplemented with 10% (*v*/*v*) FBS. Both cell types were cultured at 37 °C in a humidified incubator with 5% CO_2_. MTT reagent (0.5 mg/mL) was added to the wells of the cell culture plate and incubated at 37 °C for 0.5 h to check cell viability. DMSO was added to dissolve the purple crystals. The OD absorbance of each sample was measured at 540 nm.

### 4.3. Measurement of NO Generation

NO synthesis was analyzed by measuring nitrite (NO_2_^−^) accumulation in BV2 cell culture supernatant using the Griess method. The Griess solution and an equivalent amount of culture supernatant were mixed together. A microplate spectrophotometer was used to measure absorbance at 570 nm. Nitrite concentration was determined according to the standard curve (0, 1, 5, 10, and 20 µM) generated using sodium nitrite (NaNO_2_) solution. The average absorbance of each sample was measured and their concentrations were determined by comparing them with the standard curve.

### 4.4. ELISA

The concentrations of inflammatory factors, including IL-6, PGE_2_, and TNF-α, in cell culture supernatant were measured using the corresponding mouse ELISA Kit, according to the manufacturer’s instructions.

### 4.5. Western Blot Analysis

Cells were lysed with RIPA buffer supplemented with a mixture of protease and phosphatase inhibitors. After incubation on ice for 15 min, centrifuged and stored at −80 °C, cytoplasmic and nuclear proteins were isolated from cells using commercial kits for NF-κB pathway. The protein concentration in the lysate was determined using a protein assay kit. Cell-containing lysates were separated by SDS-PAGE. Proteins were transferred to an NC membrane and then blocked in 5% skim milk for 60 min. The membranes were incubated with the primary antibody overnight at 4 °C. The secondary antibody was horseradish peroxidase-conjugated anti-IgG, which was incubated with the membrane for 1 h at room temperature. Immune complexes were visualized using an enhanced chemiluminescence system, and the optical density of the bands was determined using ImageJ 6.0 software.

### 4.6. NF-κB (p65) Localization

The cell supernatant was removed from the cell culture plate, and the cells were inoculated and cultured on the Lab Tek II slides. Cells were washed with PBS and then fixed with 10% formalin. The cells were then incubated with specific p65 antibodies and Alexa Fluor 488. DAPI was used to stain the nuclei. Finally, the cells were observed and photographed under a fluorescence microscope (Provis AX70; Olympus Optical Co., Tokyo, Japan).

### 4.7. Determination of ROS Levels in HT22 Cells

Intracellular ROS production was measured using DCFDA fluorescent probes. Cells were treated with 10 μM DCFDA at 37 °C for 30 min. Next, the DCFDA solution was removed, and cells were washed with PBS. The cells were observed under a fluorescence microscope and ImageJ 6.0 software was used to analyze the fluorescence intensity.

### 4.8. Statistical Analysis

GraphPad Prism 5.0 software was used to perform one-way ANOVA on all values, which were displayed as mean ± SD. Statistical significance was determined at *p* < 0.05.

## 5. Conclusions

Dihydropashanone inhibits neurotoxin-induced neuroinflammation via reducing NF-κB activation in microglia and has a protective effect against glutamate-mediated neuronal toxicity. Dihydropashanone exerts its antioxidant effects via the Nrf2/HO-1 signaling pathway. In conclusion, dihydropashanone offers potential for broad clinical application in the treatment of neurodegenerative illnesses such as AD.

## Data Availability

Data supporting the findings of this study are available upon request from the corresponding author.
